# A panel of blood-based circulatory miRNAs with diagnostic potential in patients with psoriasis

**DOI:** 10.3389/fmed.2023.1207993

**Published:** 2023-08-28

**Authors:** Priyanka Madaan, Uttam Sharma, Nipanshi Tyagi, Balvinder Kaur Brar, Shivani Bansal, Hemant Rituraj Kushwaha, Harmanpreet Singh Kapoor, Aklank Jain, Manju Jain

**Affiliations:** ^1^Department of Biochemistry, Central University of Punjab, Bathinda, Punjab, India; ^2^Department of Zoology, Central University of Punjab, Bathinda, Punjab, India; ^3^School of Biotechnology, Jawaharlal Nehru University, New Delhi, India; ^4^Department of Skin and VD, Guru Gobind Singh Medical College and Hospital, Faridkot, Punjab, India; ^5^Department of Dermatology, All India Institute of Medical Sciences, Bathinda, Punjab, India; ^6^Department of Mathematics and Statistics, Central University of Punjab, Bathinda, Punjab, India

**Keywords:** psoriasis, diagnostics, circulatory miRNAs, miR-215, miR-148a, miR-223, miR-125b, miR-142-3p

## Abstract

Psoriasis is a chronic inflammatory skin disease with keratinocyte hyperproliferation and T cells as key mediators of lesional and systemic inflammatory changes. To date, no suitable differential biomarkers are available for the disease diagnosis. More recently, microRNAs have been identified as critical regulators of lesional and systemic immune changes in psoriasis with diagnostic potential. We have performed expression profiling of T cell-specific miRNAs in 38 plasma samples from psoriasis vulgaris patients and an equal number of age- and gender-matched healthy subjects. Our findings have identified a panel of five blood-based circulatory miRNAs with a significant change in their expression levels, comprising miR-215, miR-148a, miR-125b-5p, miR-223, and miR-142-3p, which can differentiate psoriasis vulgaris patients from healthy individuals. The receiver operating characteristic (ROC) curves for all five miRNAs individually and in combination exhibited a significant disease discriminatory area under the curve with an AUC of 0.762 and a *p* < 0.0001 for all the miRNAs together. Statistically, all five miRNAs in combination depicted the best-fit model in relation to disease severity (PASI) compared with individual miRNAs, with the highest R^2^ value of 0.94 and the lowest AIC score of 131.8. Each of the miRNAs also exhibited a significant association with at least one of the other miRNAs in the panel. Importantly, the five miRNAs in the panel regulate one or more immune-inflammation pathways based on target prediction, pathway network analysis, and validated roles in the literature. The miRNA panel provides a rationalized combination of biomarkers that can be tested further on an expanded cohort of patients for their diagnostic value.

## Introduction

Psoriasis is a chronic inflammatory skin disease with itchy and painful cutaneous manifestations that affects nearly 2% of the world's population (1, 2). Psoriatic lesions are characterized by abnormal differentiation and hyperproliferation of keratinocytes with immune cell infiltration. Disease etiology involves a complex interplay between genetic and environmental factors that disturbs the homeostatic cross-talk between the skin keratinocytes and different immune cells normally observed in healthy skin. Despite substantial studies on the initiation and progression of the disease, the exact molecular mechanisms that dysregulate the complex interactions among the lesional cells to create a chronic inflammatory environment are not fully known. Also, the significance of systemic immune changes associated with the disease is not well understood (1). Immunologically, new insights into the pathogenesis of psoriasis imply a major role of T cells in the initiation and maintenance of the inflammatory state, which can potentially lead to keratinocyte-specific changes observed in the disease lesions (3, 4). Generally, the disease is diagnosed by clinical evaluation of skin lesions by expert dermatologists, with occasional histopathological examination (5). With no reliable molecular biomarkers as criteria for clinical diagnostics to date, there is an urgent requirement for differential biomarkers with diagnostic potential and predictive value.

Psoriasis is the result of several genetic–epigenetic, environmental, and immunological factors, of which miRNAs have recently emerged as critical regulators of disease pathogenesis (6–11). miRNAs are small (~20–25 bp) non-coding RNAs that control gene expression in a cell-specific manner with wide functional implications in processes such as development, growth, apoptosis, plasticity, activation, survival, proliferation, and differentiation (12, 13). In this context, miRNAs that regulate keratinocyte and heterogeneous T cell populations become significant in the pathophysiology of psoriasis (12–16). Although produced within cells, miRNAs with altered cellular expression can potentially mirror dysregulated stable cell-free molecules in peripheral circulation under disease conditions (17, 18). Thus, a disease-specific circulatory miRNA pool makes it amenable to developing minimally invasive blood-based diagnostic biomarkers in relation to disease prediction and severity. Several studies on miRNA expression analysis have been carried out across various sample types, viz., blood, PBMCs, hair, and lesional skin, for the potential diagnosis of psoriasis (19–22). Explorations of differentially expressed systemic miRNAs in sera/plasma samples with select miRNA candidates have been carried out in a few studies based on human miRNA arrays and small RNA sequencing analysis (19, 20, 22–27). Heterogeneous findings on miRNA candidates with respect to disease-specific differential expression, low abundance, a non-significant correlation with disease severity, a lack of knowledge on the role of altered miRNAs in disease progression, a small sample size used, and variation in blood components used as samples in these studies are limiting the transition to their clinical use.

In the present study, a set of immunologically relevant, T cell-specific miRNA candidates has been selected to access their differential expression in the plasma of psoriatic patients (28–35). With the potential to drive inflammatory disease processes, we have deciphered a combination of five significant miRNAs that can provide a robust diagnostic panel in light of their functional implication in disease etiology and progression.

## Materials and methods

### Study subjects and sample collection

A total of 40 psoriasis vulgaris patients clinically confirmed by dermatologists along with 40 gender-matched healthy subjects were enrolled in the study. The patients with psoriasis were recruited from the outpatient dermatology clinic at the Guru Gobind Singh Medical College and Hospital, Faridkot, Punjab, India, and the All India Institute of Medical Sciences, Bathinda, Punjab, India. Patients aged 18 years or older, those with a clinical diagnosis of mild-to-severe psoriasis vulgaris (PV), those who have not received topical therapy for at least 2 weeks, and those who have been receiving systemic immunosuppressive treatment or phototherapy for at least 1 month were included, while psoriasis patients with other infections or co-morbidities were not enrolled in the study. Disease severity was graded by the psoriasis area and severity index (PASI) score and body surface area (BSA) into mild (PASI score ≤ 5/BSA < 3%), moderate (PASI score 5–10/BSA 3–10%), and severe (PASI score >10/BSA> 10%) (36); 5-mm punch biopsies were taken from the lesional skin of 16 patients. Non-lesional biopsies from the same 16 patients from the uninvolved skin away from the lesion were also collected for comparative analysis. Healthy age- and gender-matched controls were recruited from the staff and graduate students at the Central University of Punjab, Bathinda, Punjab, India. Written informed consent, along with a subject information form, was obtained from all the participants. The present study was conducted in accordance with the Declaration of Helsinki and approved by the Ethics Committees of Guru Gobind Singh Medical College and Hospital, Faridkot, Punjab, India (GGS/IEC/19/57), AIIMS, Bathinda, India (IEC/AIIMS/BTI/150), and Central University of Punjab, Bathinda, Punjab, India (CUPB/IEC/2016/045).

### Sample processing

Of the 40 patients enrolled in the study, lesional and non-lesional tissues from 16 psoriasis patients were processed for disease-specific histopathological examination, and 5-mm lesional and non-lesional punch biopsies were collected and fixed in 10% neutral buffered formaldehyde (NBF). Paraffin-embedded tissue was processed into 5 μm sections and stained with hematoxylin and eosin (H&E) for histopathological analysis. Whole blood samples from 40 psoriasis patients and 40 healthy controls were collected in EDTA-coated vials. The blood samples were centrifuged at 1,800 rpm for 10 min at RT to collect the straw-colored platelet-rich plasma. The plasma was centrifuged again at 2,000 rpm for 10 min at 4°C to remove the remaining cellular components. The purified plasma samples were stored in aliquots at −80°C until further assays and 200 μl of each plasma sample was used for miRNA extraction.

### Circulating miRNA isolation, cDNA synthesis, and qRT-PCR assays

miRNA extraction from plasma samples was performed using a miRNeasy serum/plasma kit (Catalog No. 217184: Qiagen, Inc., USA) according to the manufacturer's instructions. The concentration and purity of miRNA were determined with a NanoDrop UV-Vis Spectrophotometer 2000cc (Thermo Fisher Scientific, CA, USA), and 100 ng of total isolated miRNA was used for cDNA synthesis, using the miScript II RT kit following the manufacturer's protocol (Catalog no. 218161: Qiagen, Inc., USA). A set of 15 immunologically relevant miRNAs with respective sequences enlisted in Supplementary Table S1 were quantified in plasma samples of psoriasis patients compared with a healthy cohort by real-time PCR using the Qiagen miScript SYBR Green PCR Kit and miR-specific primers (Catalog no. 218073: Qiagen, Inc., USA, Cat. no. MS00004900; Qiagen, Inc., QuantStudio 3 Applied Biosystems real-time PCR). Cel-miR-39 was used as an internal control. qRT-PCR comprised 6.25 μl of SYBR Green Master Mix, 1.25 μl each of miR-specific primer and universal primer, and 3.5 μl of nuclease-free water with the required amount of cDNA. The reaction conditions were as follows: initial activation was performed at 95°C for 15 min, denaturation at 94°C for 15 s, annealing at 55°C for 30 s, and extension at 70°C for 30 s for 40 cycles with melting curve analysis for quality check. A non-template control reaction for each primer was also carried out to check for contamination by including all components except the cDNA template. The threshold cycle (Ct) value for each sample with primers was recorded. All samples were tested at least three times. The relative expression between psoriasis patients and healthy controls was estimated by the 2^−ΔCt^ method (37, 38). Briefly, the miRNA-specific Ct values for each of the psoriasis and control samples were normalized using the respective Ct values for Cel-miR-39 as a spike-in control. The average of normalized Ct values for the psoriasis group and control group was further used for the calculation of fold changes for each miRNA tested.

### Network analysis of miRNA-target pathways

Target genes for five miRNAs of interest, viz., miR-215, miR-142-3p, miR-223, miR-148a, and miR-125b-5p, were predicted using four different miRNA-target prediction tools: miRDB (http://www.mirdb.org/), TargetScan (https://www.targetscan.org/vert_80/), DIANA-microT (https://dianalab.e-ce.uth.gr/html/dianauniverse/index.php?r=microT_CDS), and Mirabel (http://bioinfo.univ-rouen.fr/mirabel/). For each miRNA, common targets were deciphered based on the four databases. The Kyoto Encyclopedia of Genes and Genomes (KEGG) pathways were enriched for each miRNA using Starbase (https://starbase.sysu.edu.cn/). The enriched pathways were analyzed for the signaling components that overlapped with the common set of targets for each miRNA retrieved from the four databases. Significant pathways involving miRNA-specific targets were reviewed for potential roles in T cells and/or keratinocyte-associated biological functions and were mapped for interaction networks using Cytoscape.

### Statistical analysis

The non-parametric Mann–Whitney U-test was performed to compare the qRT-PCR-based miRNA expression differences in the psoriasis patient group and the age- and gender-matched healthy control group. Furthermore, 2^−ΔCt^ values were used to generate receiver operating characteristic (ROC) curves with a calculation of the area under the curve (AUC) as an index for evaluating the diagnostic potential of miRNAs of interest to differentiate psoriasis patients from healthy controls (39). A *p* ≤ 0.05 was considered statistically significant for qRT-PCR data and ROC analysis. All statistical calculations were performed using GraphPad Prism 8.0.2 and R software 4.2.2. Pearson correlation coefficients were derived to study the pairwise relationship between the expression levels of each of the miRNAs among study subjects (psoriasis patients and control subjects). A *p* ≤ 0.05 was considered statistically significant. A non-linear model b-spline was used to study the individual and the joint impact of miRNA levels in relation to the PASI score.

## Results

### Study cohort

**Figure 1:**
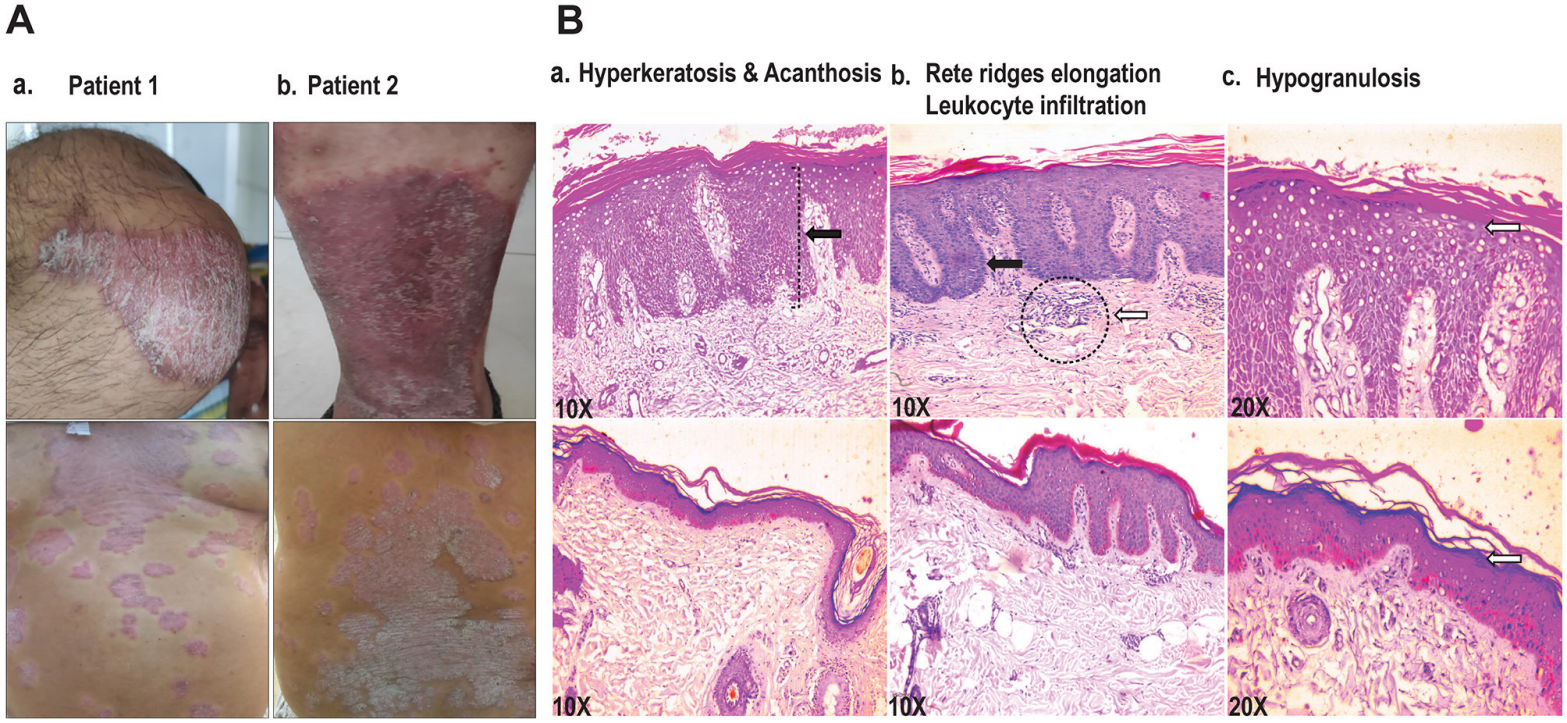
**(A)** Representative images of psoriasis patients showing well-demarcated scaly, erythematous lesions. **(B)** Representation of histopathologic features as observed in hematoxylin- and eosin-stained sections of lesional and non-lesional tissue from psoriasis vulgaris patients. a. Hyperkeratosis and acanthosis (black arrow), original magnification x10. b. Extension of rete ridges (black arrow) and leukocytic dermal infiltration (dashed circle with white arrow) in lesional tissue as compared to the non-lesional tissue, original magnification x10 c. Reduced granular layer (hypogranulosis) in lesional tissue, original magnification ×20.

A total of 40 patients clinically confirmed for psoriasis vulgaris and 40 healthy subjects enrolled in the study were grouped into age- and gender-matched study cohorts. Patients including men and women comprised clinically identifiable individuals with typical dry, raised, erythematous psoriatic plaques exhibiting silvery scales and were graded as mild, moderate, and severe based on disease severity, with the PASI score ranging from 3.5 to 15.6 and the BSA from 2% to 58% (Figure 1A, Table 1); 5 mild, 18 moderate, and 17 severe psoriasis patients with no ongoing treatment at the time of diagnosis and sample collection were enrolled in experimental studies. Histopathological analysis of lesional vs. non-lesional tissue was performed on biopsies from 16 patients. Hematoxylin and eosin-stained skin lesional sections exhibited psoriasis-specific dermal and epidermal changes to variable extents (Figure 1B, Table 1). All 16 patients exhibited thickening of the epidermis designated as acanthosis, with severe manifestations leading to elongation of rete ridges in 12 of the 16 patients. Hyperkeratosis with thickened stratum corneum, parakeratosis with abnormal retention of nuclei in the stratum corneum layer, and dermal leukocyte infiltrates were apparent in 13 specimens with hypogranulosis in 12 patients. An exploration of the systemic expression profile of miRNAs of interest was further carried out in the study cohorts.

**Table 1:** Baseline characteristics of psoriasis vulgaris (PV) patients and healthy controls (HC).

**Baseline characteristics of controls and psoriasis patients**									**Histopathological characteristics (H&E examination)**
**PV patients and healthy controls**	**Age groups**					**PASI score/BSA**			**No. of patients (*****n*** = **16) With psoriasis features**
PV patients(*n* = 40)	18-30	30-40	40-50	50-60	>60	Mild(n=5/40)(PASI < 5, BSA < 3%)	Moderate (n=18/40) (PASI 5-10, BSA 3-10%)	Severe (n=17/40)(PASI>10,BSA >10%)	Acanthosis: 16; Parakeratosis: 13; Hyperkeratosis: 13; Leukocytes infiltration: 13; Elongated rete ridges: 12; Hypogranulosis: 12
Men (28/40)	7	8	3	7	3	4	14	10	
Women (12/40)	4	1	3	2	2	1	4	7	
Controls (*n* = 40)									
Men (28/40)	9	8	2	5	4				
Women (12/40)	6	1	2	1	2				

### Expression pattern of select plasma miRNAs

**Figure 2:**
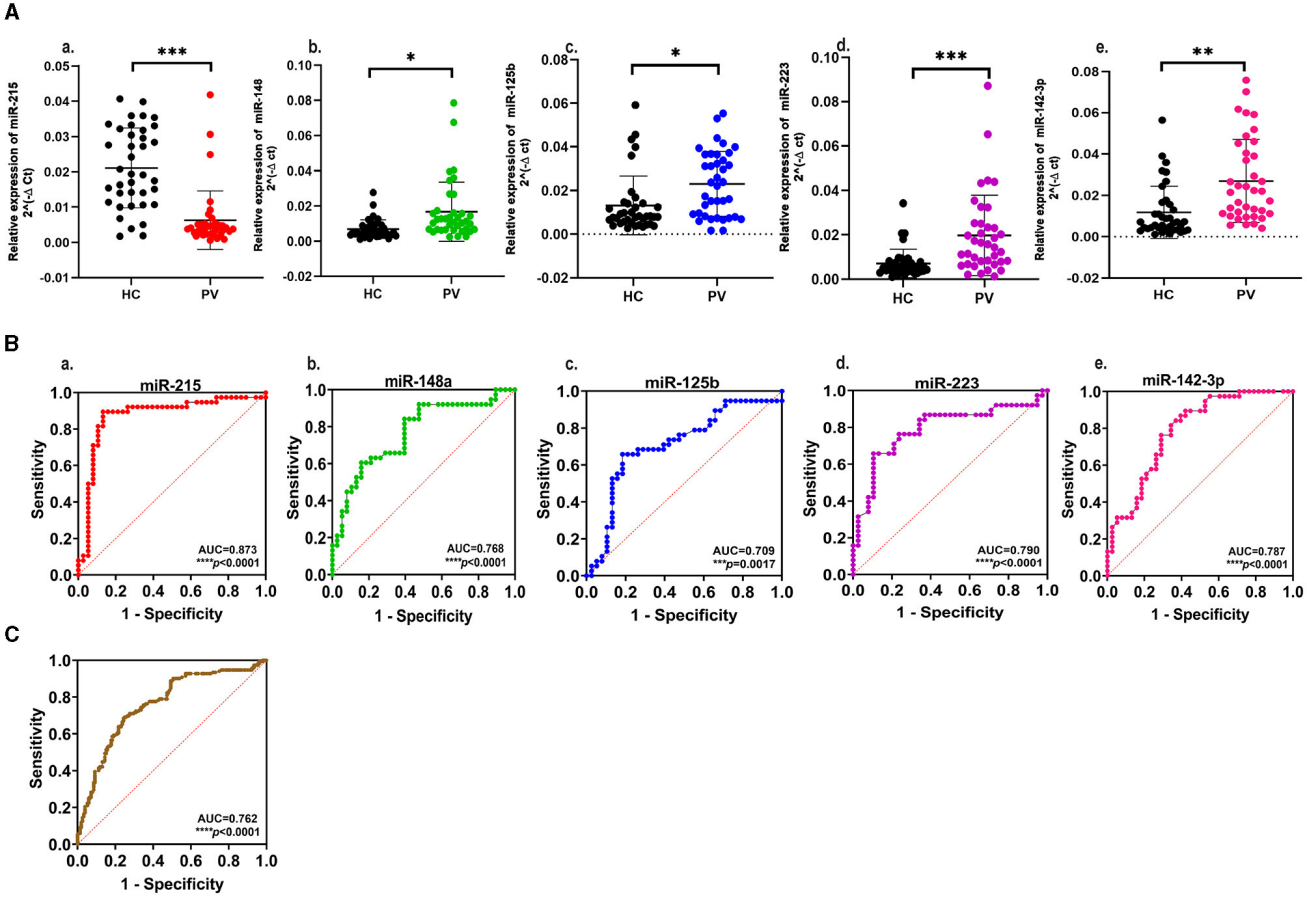
**(A)** Expression profiling of select miRNAs in plasma samples from psoriasis vulgaris patients (PV) in comparison to healthy controls (HC): a. miR-215; b. miR-148a; c. miR-125b; d. miR-223; e. miR-142-3p. The scatter plot represents normalized 2^−ΔCt^ values of individual samples. Black dots represent healthy controls, and PV patients are represented by red dots for miR-215, green dots for miR-148a, blue dots for miR-125b, purple dots for miR-223, and pink dots for miR-142-3p. Data are expressed as mean ± SD. ***p < 0.001, **p < 0.01, and *p < 0.05 calculated using the Mann–Whitney U-test. Data represent three independent qRT-PCR experiments. **(B)** Receiver operating characteristic (ROC) curve with area under the curve showing the diagnostic value of the select miRNAs a. miR-215; b. miR-148a; c. miR-125b; d. miR-223; e. miR-142-3p; ****p < 0.0001 and ***p <0.001. **(C)** Receiver operating characteristic (ROC) curve showing the significant diagnostic value of five miRNAs in combination, ****p < 0.0001.

A set of 15 immunologically relevant miRNA candidates were explored for qRT-based differential expression in the plasma samples of 12 psoriasis patients and 12 age- and gender-matched healthy controls (Supplementary Table 1). In total, five miRNAs, viz. miR-215, miR-148a, miR-125b, miR-223, and miR-142-3p, exhibited an expression pattern with consistent and significant changes. miR-215 was significantly decreased (fold change = −2.24, *p* = 0.043), while four miRNAs miR-148a, miR-125b, miR-223, and miR-142-3p were significantly upregulated (fold change = 1.82, 1.84, 2.42, and 2.56 and *p* = 0.032, 0.034, 0.028, 0.045, respectively) in psoriasis patients compared with healthy controls. miR-146a and miR-21 showed a trend toward upregulation with no significant change, while no change was observed in miR-155 expression levels. The remaining miRNA candidates, miR-590-5p, miR-15b, miR-568, miR-150, miR-23b, miR-27b, and miR-184, were associated with high sample-to-sample variation in terms of threshold detection or low abundance. The five miRNAs with significant dysregulation in expression levels were further validated in an extended cohort of psoriasis patients and healthy study subjects. In line with initial results, cumulative study cohorts with 38 psoriasis patients versus 38 healthy subjects exhibited significant downregulation in miR-215 expression (fold change = −3.34, *p* < 0.001) and significant upregulation in miR-148a (fold change = 2.43, *p* < 0.05), miR-125b (fold change = 1.75, *p* < 0.05), miR-223 (fold change = 2.79, *p* < 0.001), and miR-142-3p (fold change = 2.27, *p* < 0.01), as shown in Figure 2A and Table 2. The five miRNAs of significance were tested for their diagnostic potential to differentiate psoriasis vulgaris patients from healthy individuals.

**Table 2:** Differential gene expression and receiver operating curve (ROC) analysis with the area under the curve (AUC) for the expression of miRNAs in plasma samples from psoriasis vulgaris patients and healthy controls.

**Differential expression and ROC curve analysis**	**miR-215**	**miR-148a**	**miR-125b**	**miR-223**	**miR-142-3p**
Fold change	−3.34	2.43	1.75	2.79	2.27
(*p-*value)	0.0002 (^***^)	0.0284 (^*^)	0.0441 (^*^)	0.0006 (^***^)	0.0043 (^**^)
AUC	0.873	0.768	0.709	0.790	0.787
(*p-*value)	< 0.0001 (^****^)	< 0.0001 (^****^)	0.0017 (^***^)	< 0.0001 (^****^)	< 0.0001 (^****^)
SE	0.0463	0.0546	0.0617	0.0546	0.0520
95% CI	0.782–0.963	0.661– 0.875	0.588–0.829	0.683–0.897	0.685–0.889

^****^p ≤ 0.0001, ^***^p ≤ 0.001, ^**^p ≤ 0.01, ^*^p ≤ 0.05; fold change with negative sign represents downregulated miRNA expression.

### miR-215, miR-148a, miR-125b, miR-223, and miR-142-3p constitute a potential diagnostic panel for psoriasis vulgaris

The receiver operating characteristic (ROC) curves were generated for each of the significantly altered miRNAs, and the area under the receiver operating characteristic curve (AUC) was employed as an accuracy index to evaluate the diagnostic performance of the five miRNAs with significant expression changes. The ROC analysis of miR-215, miR-148a, miR-125b, miR-223, and miR-142-3p corresponded to AUCs of 0.873, 0.768, 0.709, 0.790, and 0.787, respectively, indicating a robust discriminatory potential of each of the miRNAs (Figure 2B, Table 2). The combination of all five miRNAs together also showed significant disease discrimination readout with an AUC of 0.762 and a *p* < 0.0001 (Figure 2C).

**Figure 3:**
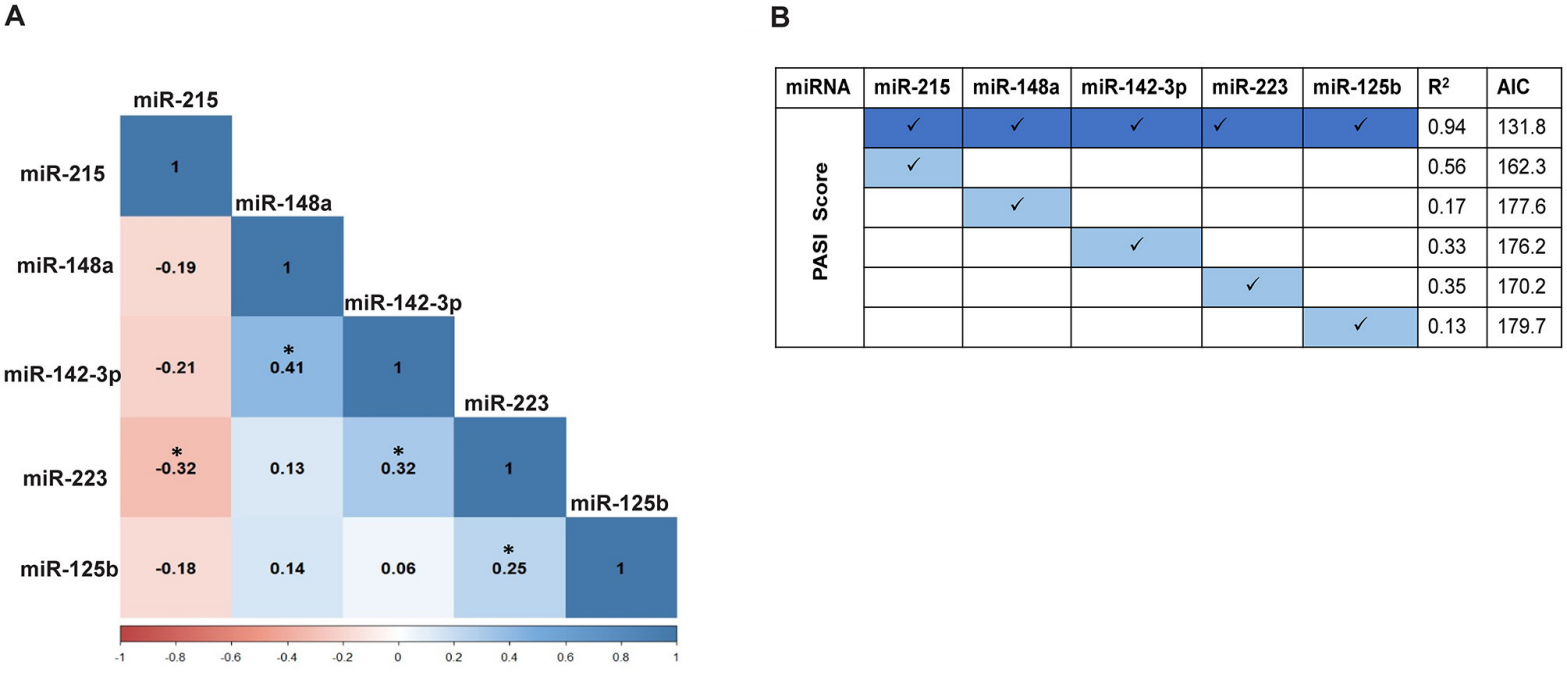
**(A)** Pairwise correlation between the expression levels of the five miRNAs using Pearson correlation. The values shown depict the correlation coefficient (r) with a significant correlation indicated by *p ≤ 0.05. **(B)** Correlation analysis of miRNA expression levels with PASI score using the b-spline model. All five miRNAs together exhibit the best-fit model with a coefficient of determination, R^2^ = 0.94, Akaike information criterion (AIC) = 131.8. The b-spline model for individual miRNAs with PASI scores does not exhibit a good-fit model.

A correlation analysis between each of the five miRNAs for the control and psoriasis subjects was performed to explore the association between their expression patterns (Figure 3A, Table 3). Based on the Pearson correlation coefficient as a readout, each of the five miRNAs exhibited a significant correlation with at least one of the miRNAs in the panel. miR-215 showed a negative correlation with all other 4 miRNAs and a significant association only with miR-223 (*p* < 0.05). miR-148a, miR-142-3p, miR-223, and miR-125b showed a trend toward a positive correlation between each other with significant positive correlations between miR-148a and miR-142-3p (*p* < 0.05), miR-142-3p and miR-223 (*p* < 0.05), and miR-223 and miR-125b (*p* < 0.05). The AUC values along with the correlation matrix imply that the miRNA panel with the combination of a downregulated miR-215 and upregulated miRNAs miR-148a, miR-125b, miR-223, and miR-142-3p is a promising set of diagnostic miRNA biomarkers for psoriasis vulgaris patients.

**Table 3:** Pairwise correlation between miRNAs represented as Pearson correlation coefficient **(r)** with *p-values* given in parentheses.

**miRNAs**	**miR-215**	**miR-148a**	**miR-125b**	**miR-142-3p**	**miR-223**
miR-215	1	−0.19 (0.1030)	−0.18 (0.1297)	−0.21 (0.0658)	−0.32^*^ (0.0049)
miR-148a	−0.19 (0.1030)	1	0.14 (0.2131)	0.41^*^ (0.0002)	0.13 (0.245)
miR-125b	−0.18 (0.1297)	0.14 (0.2131)	1	0.06 (0.6185)	0.25^*^ (0.0305)
miR-142-3p	−0.21 (0.0658)	0.41^*^ (0.0002)	0.06 (0.6185)	1	0.32^*^ (0.0051)
miR-223	−0.32^*^ (0.0049)	0.13 (0.2451)	0.25^*^ (0.0305)	0.32^*^ (0.0051)	1

The ^*^ symbol indicates the value of *p* < 0.05.

### Correlation of miRNA expression with clinical severity

All five miRNAs were further analyzed for their correlation with disease severity in terms of patient PASI score, alone or in combination. With a non-linear association pattern derived between miRNA expression levels and PASI scores, the b-spline model was used to decipher their relationship. Based on the best-fit b-spline model, all five miRNAs together exhibited the best-fit model with an R^2^ value = 0.94 and the lowest AIC score of 131.8 (Figure 3B). None of the individual miRNAs showed a good fit with the PASI score, with lower R^2^ values and higher AIC readouts. Individually, miR-215 alone showed the highest correlation (R^2^ value = 0.56 and AIC = 162.3) compared with each of the other miRNAs. The additive effect derived from the b-spline model in terms of R^2^ and AIC values with all five miRNAs taken together implies a robust correlation with disease severity, unlike individual miRNAs.

### Understanding candidate miRNA-target pathways with the functional implication in psoriasis

**Figure 4:**
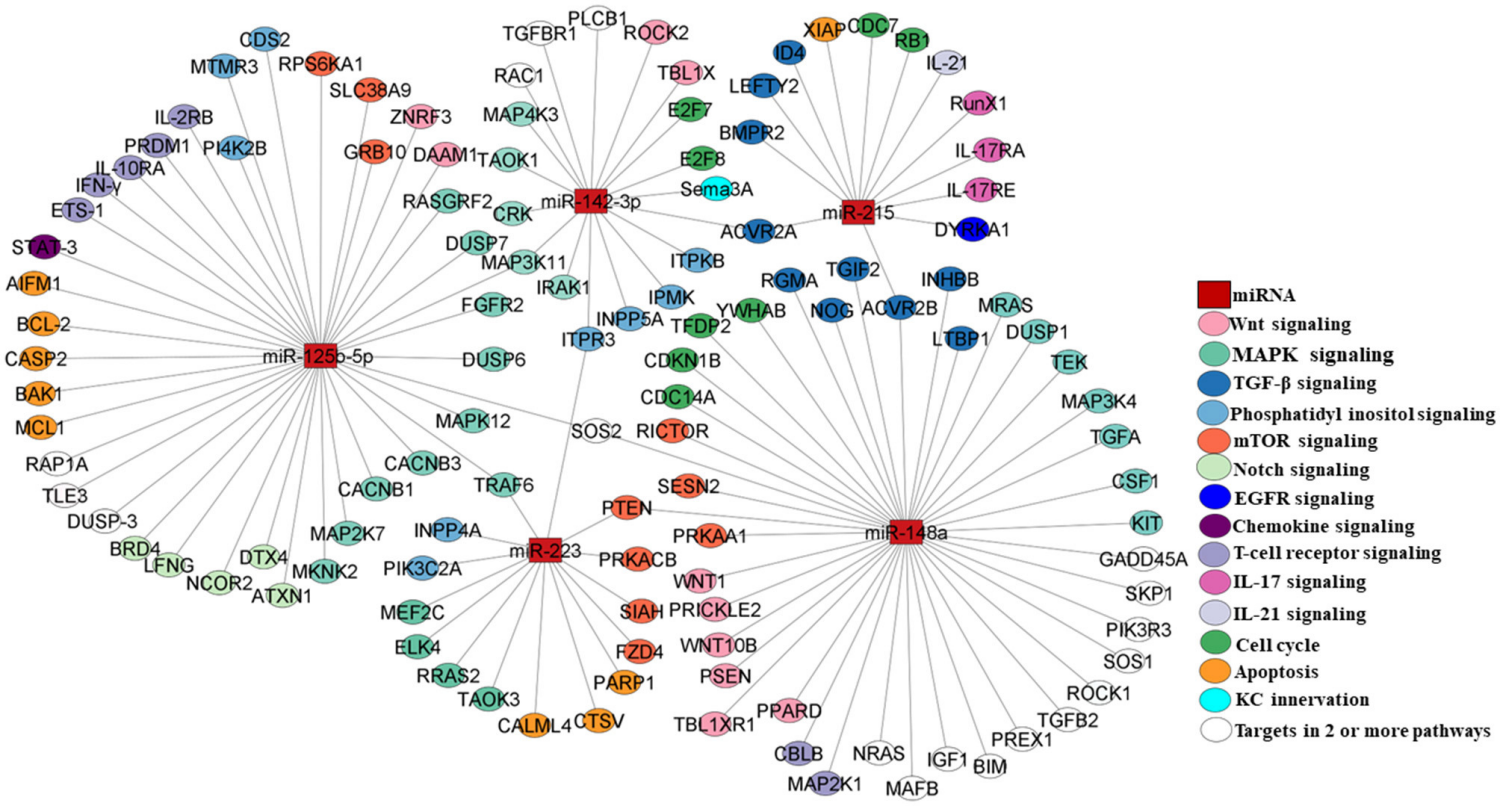
miRNA–mRNA network mapping for miR-215, miR-148a, miR-223, miR-125b, and miR-142-3p as select miRNAs of interest with their direct targets (please see Table 4). miRNAs are depicted as rectangular boxes, and their targets are represented as circles. The color coding of the targets represents their functionality in the corresponding pathways as highlighted in different colors. Targets in empty circles represent those candidates involved in two or more pathways.

Enrichment of significant pathways involving direct targets of the dysregulated panel of five miRNAs, i.e.,miR-215, miR-142-3p, miR-125b, miR-148a, and miR-223, was performed using a combination of the miRNA-specific targets and KEGG pathways as detailed in the Materials and Methods section. A set of signaling pathways downstream of one or more than one miRNA were delineated in light of the literature as shown in Table 4. All the relevant targets involved in the enriched pathways were mapped as a miRNA–mRNA regulatory network. The panel of five dysregulated miRNAs exhibited a network of interconnected targets as part of one or more enriched pathways of interest, which is color-coded in Figure 4 with supporting information in Table 4. Each of the pathways was found to be relevant to the pathophysiology of psoriasis based on their validated or predicted role in T cell and/or keratinocyte biology. miR-142-3p, miR-125b, miR-148a, and miR-223 are upstream of multiple components of Wnt signaling implicated in keratinocyte proliferation and apoptosis in psoriatic lesions, along with a key role in T cell development and differentiation of peripheral T cell subsets (40, 41, 71–74). With a role in vast cellular functions, MAPK signaling enriched with targets specific for miR125b and miR-223, miR-142-3p, and miR-148a is known to be involved in keratinocyte proliferation and differentiation with a multidimensional role in T cell activation, differentiation, and effector function (42–44, 71–74).

**Table 4:** Select miRNAs with their targets and associated signaling pathways.

**Relevant miRNA-target Pathways (KEGG pathways+ literature)**	**miRNAs of interest with their validated/predicted targets**					**Functional role in psoriasis (validated/predicted)**		**References**
**miRNAs KEGG pathways**	**miR-215**	**miR-142-3p**	**miR-125b**	**miR-223**	**miR-148a**	**Keratinocytes (KC)**	**T cells**	
WNT signaling pathways		TBL1X, ROCK2, RAC1, PLCB1	ZNRF3, TLE3, DAAM1	SIAH 1, FZD4, APC, PRKACB	SKP1, TBL1XR1, PRICKLE2, PPARD, WNT1, WNT10B, PSEN	Keratinocyte proliferation and apoptosis	T cell development and differentiation	(40, 41)
MAPK signaling pathways		TGFBR1, RAC1, IRAK1, MAP4K3, MAP3K11, TAOK1, CRK	FGFR2^V^, CACNB1, CACNB3, RASGRF2, RAP1A, FGFR2, MKNK2, SOS2, RPS6KA1, TRAF6, MAP3K11, DUSP7, MAP2K7, DUSP6 MAPK12	IGF1R, RRAS2, ELK4, TAOK3, MEF2C	TGFA, IGF1, CSF1 KIT, TEK, SOS1, SOS2, NRAS, MRAS, TGFB2 GADD45A MAP3K4, DUSP1	Proliferation and differentiation of KCs. miR-125b shown to modulate KC proliferation via FGFR2	T cell activation and differentiation and effector functions	(42–44)
TGF-β signaling	ACVR2A, ACVR2B, LEFTY2, ID4, BMPR2	ACVR2A, TGFBR1			ACVR2B, INHBB, LTBP1, NOG, RGMA, ROCK1, SKP1, TGFB2, TGIF2	KC hyperproliferation	T cell homeostasis	(45, 46)
Phosphatidylinositol signaling pathway		PLCB1, IPMK ITPKB, INPP5A, ITPR3	PI4K2B, MTMR3, CDS2	ITPR3, CALML4, PIK3CA, INPP4A, PTEN ^V^		Proliferation of KCs. miR-223 regulates KC proliferation via PTEN	T cell activation and differentiation	(47–49)
mTOR signaling pathway			SLC38A9, GRB10 EIF4, EBP1, PRKAA1, DVL3		SESN2, PRKAA1, IGF1, SOS1, SOS2, NRAS, PIK3R3, PTEN, RICTOR	Regulates hyperproliferation and aberrant differentiation in psoriatic lesions	T cell proliferation and metabolism	(50, 51)
EGFR signaling	DYRKA1^V^					Regulates proliferation and inflammation. miR-215 inhibits proliferation via DYRKA1.	Not studied in the context of T cells in Psoriasis	(52)
KC innervation		Sema3A^V^				Regulates KCs miR-142-3p alter KC proliferation and apoptosis via Sema3A	Not studied in the context of T cells in psoriasis	(53)
Notch signaling			BRD4^V^, LFNG DTX4, TLE3 NCOR2, ATXN1			Regulates KC differentiation. miR-125b regulates cell proliferation via BRD4 in Notch signaling	Regulates T cell development, activation and effector function	(54, 55)
Chemokine signaling pathway			STAT3^V^, RAP1A		SOS1, SOS2, NRAS, PIK3R3, ROCK1, PREX1	KC-induced inflammation miR-125 regulates KC proliferation via STAT3	T cell-induced immune-inflammation	(56, 57)
T cell receptor signaling pathway			MAPK12, MAP2K7, IFN-γ^V^ IL-2RB^V^, IL-10RA^V^, PRDM1^V^		SOS1, SOS2, MAP2K1, PIK3R3, CBLB	Not relevant in KCs	T cell activation and homeostasis. miR-125b maintains T cell naivety	(30, 58, 59)
IL-21 signaling	IL-21^V^					Epidermal hyperplasia	T cell activation, proliferation, and differentiation. miR-215 targets IL-21	(60–62)
IL-17 signaling	IL-17RA^V^ IL-17RE^V^ RUNX1^V^					Mediator of KC inflammatory loop and hyperproliferation	Enhanced Th17-mediated inflammation. miR-215 modulates IL-17 receptors and RunX1.	(63–65)
Cell cycle	RB1, CDC7	E2F7^V^, E2F8^V^			TFDP2, TGFB2, CDKN1B, SKP1, YWHAB, CDC14A, GADD45A	Enhanced cell cycle with hyperproliferation in psoriatic lesions	T cell proliferation miR-142-3p targets E2F7, E2F8.	(66, 67)
Apoptosis	XIAP^V^			ITPR3, PARP1, CTSV		KC survival/apoptosis	miR-215 targets XIAP with a potential role in T cell apoptosis	(68–70)

The targets listed for each of the miRNAs represent predicted and/or validated downstream signaling components along with their functional role in psoriatic keratinocytes and/or T cells.

**V** represents validated targets; the specific targets have been functionally validated as per the literature; the rest of the targets were predicted using tools mentioned in the Materials and Methods section.

TGF-β signaling involving targets of miR-142-3p, miR-148a, and miR-215 constitutes a pathway with relevance in psoriasis, affecting keratinocyte proliferation and T cell homeostasis and differentiation in response to the altered cytokine milieu (45, 46, 71, 74, 75). A phosphatidylinositol signaling network with pleiotropic upstream and downstream mediators was enriched and involves targets of miR-223, miR-125b-5p, and miR-142-3p that can mediate altered keratinocyte proliferation and T cell activation/differentiation in response to varied input stimuli (47–49, 71–73). One of the important downstream extensions of the phosphatidylinositol pathway works through mTOR signaling, which is known to regulate cellular growth, proliferation, and metabolism. In our analysis, multiple mTOR signaling complex molecules are direct targets of miR125b and miR-148 in light of the key role of the axis in abnormal keratinocyte proliferation and differentiation and modulating T cell metabolism, cell proliferation, activation, and differentiation (50, 51, 72, 74, 76). EGFR signaling, modulable via miR-215-targeted dual-specificity tyrosine phosphorylation-regulated kinase 1A (DYRKA1), is another molecular module that leads to keratinocyte hyperproliferation (52). The KC innervation pathway comprises Semaphorin 3A (Sema 3A) as a direct target of miR-142-3p, with implications for keratinocyte proliferation, apoptosis, and the production of inflammatory mediators (53). BRD4/Notch signaling works via miR-125b, resulting in altered keratinocyte proliferation (54). Notch signaling is known to play a pivotal role in T cell development, activation, proliferation, and effector function (55).

Multiple chemokines produced by keratinocytes and immunocytes may contribute to the inflammatory psoriatic lesions and systemic changes involving direct targets of miR-125b and miR-148 (56). Stat3, as a direct target of miR125b and downstream of multiple chemokine/cytokine inflammatory pathways, has been shown to dysregulate keratinocyte proliferation and apoptosis (57). T cell receptor signaling constitutes the primary event for a homeostatic effector T cell function, which is modulated in the autoimmune condition of psoriasis with a pathogenic T cell response (58, 59). The perpetual T cell activation drives the keratinocyte inflammatory loop with the manifestation of psoriatic hyperplasia (77). miR-125b-5p and miR-148 regulate multiple targets directly involved in T cell receptor signaling (72, 74). IL-21, a T cell-derived cytokine, leads to epidermal hyperplasia in psoriasis by inducing keratinocyte proliferation (60). IL-21 signaling regulates T cell activation, proliferation, and differentiation (61). Among our miRNAs of interest, miR-215 targets the IL-21 transcript (62). IL-17 signaling plays a major role in setting the inflammatory pathophysiological changes associated with psoriasis with enhanced IL-17-producing T cells and inflamed hyperproliferative keratinocytes in response to amplified IL-17 expression in psoriatic lesions and systemic circulation (63, 78). IL-17 signaling components and effector molecules, viz. IL-17RA, IL-17RE, and RUNX1, show up as direct targets of miR-215 (64, 65). Altered cell cycle progression and apoptosis in an inflammatory skin microenvironment are hallmarks of psoriatic lesions with epidermal hyperplasia (66, 68, 73–75). Specific molecules involved in these cellular processes are direct targets of miRNAs of interest. The miR-215-XIAP axis alters cellular apoptosis, while miR-142-3p targets E2F7 and E2F8, the transcription factors that regulate T cell cycling (67, 69, 70). All five miRNAs with significant expression changes in peripheral circulation showed downstream target pathways with functional roles in keratinocytes and/or T cells implying their potential role in the etiology of psoriasis.

## Discussion

Psoriasis is a cutaneous manifestation with T cells as crucial mediators of lesional and systemic inflammatory changes. The diagnosis of the disease is largely dependent on the clinical identification of typical psoriatic lesions in conjunction with histopathological findings. To date, there is a lack of reliable molecularly defined diagnostic markers for the disease, despite substantial knowledge of key cellular and molecular processes that define psoriasis. In this context, miRNAs, representing specific and stable small non-coding RNAs involved in the pathophysiology of psoriasis, comprise a promising set of molecules with diagnostic potential. A large number of such studies on the differential expression of miRNAs in varied sample types from psoriasis patients have been performed to elucidate their diagnostic and prognostic value. Limitations with respect to significant differential expression, heterogeneous abundance and correlation with disease severity, lack of knowledge on the role of altered miRNAs in disease progression, small sample test size, and variable sample types used are in the way of translating these findings to clinical use.

In the present study, 15 immune miRNAs were tested with the rationale of their functional involvement in T cell-mediated immune inflammation associated with lesional and systemic psoriatic changes. Of all the miRNAs, five candidates showed significant differential expression, with the downregulation of miR-215 and the upregulation of miR-148a, miR-223, miR-142-3p, and miR-125b-5p. All five miRNAs, individually and combinedly, exhibited promising diagnostic potential to differentiate psoriasis patients from healthy individuals as per significant and acceptable AUC values (39).

The expression pattern data of the five significant miRNAs and their correlation trend among each other and with the PASI score as a panel signify a promising combination of miRNAs with diagnostic value. The panel of five miRNAs deciphered in the present study with two newly profiled miRNAs (miR-215 and miR-148a) and three previously studied miRNAs (miR-223, miR-142-3p, and miR-125b-5p) can be tested in a larger cohort of psoriasis vulgaris patients along with a control group of patients with other skin diseases to further validate its differential diagnostic value in a clinical setting. Among the various exploratory searches for circulating diagnostic miRNAs that include global miRNA expression profiling and/or miRNA candidate-specific expression studies, this is the pilot report on a set of promising miRNAs that can be taken up as a panel for the diagnosis of psoriasis vulgaris (19, 20, 22–27).

**Figure 5:**
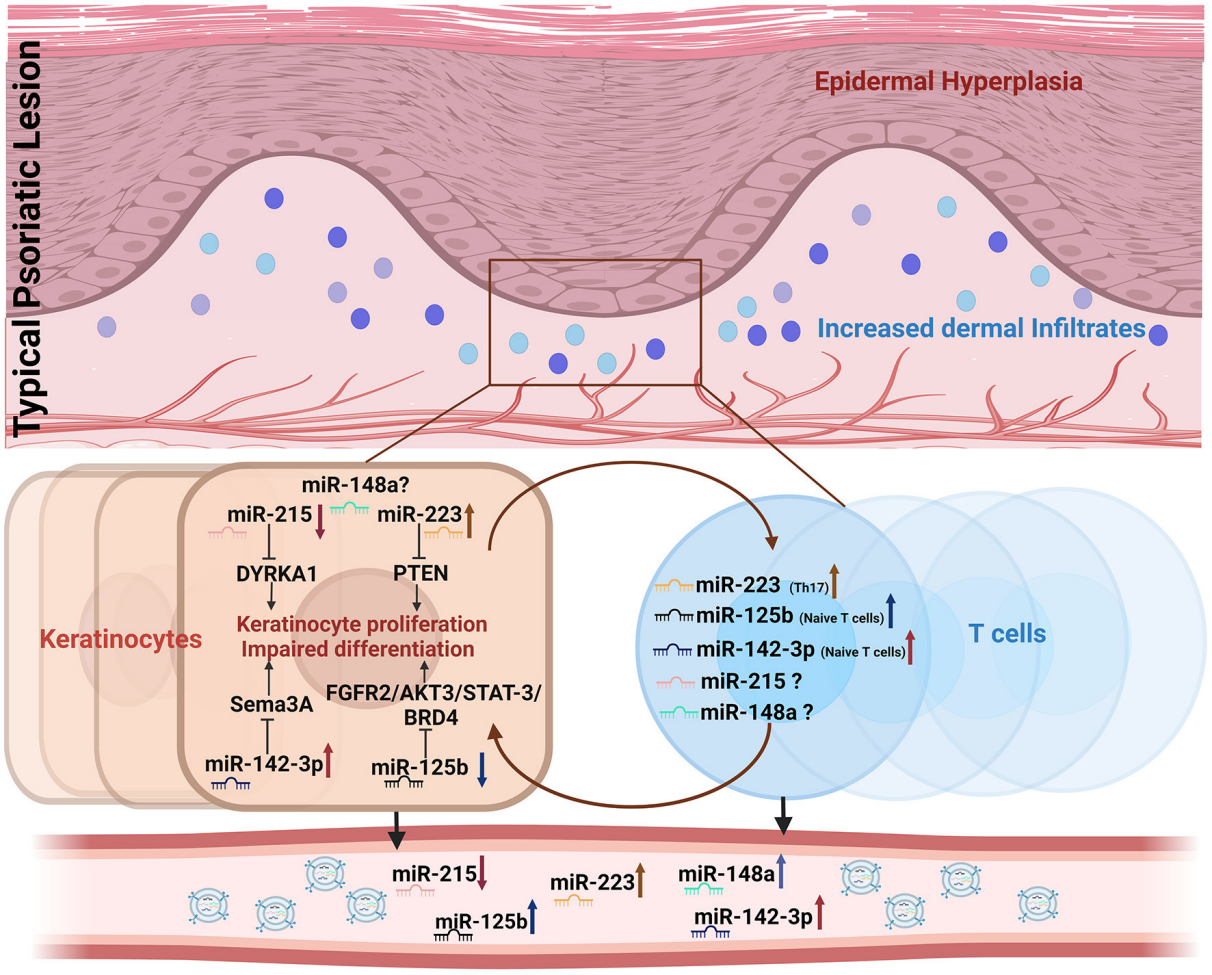
Representation of candidate miRNAs involved in keratinocyte and T cell crosstalk dysregulated in psoriasis. Dysregulated expression of miR-215, miR-148a, miR-125b, miR-223, and miR-142-3p in keratinocytes and/or T cells is shown to be altered in cell-free peripheral circulation.

Interestingly, all five miRNAs exhibited a validated and/or predicted role in dysregulating T cell and/or keratinocyte function, thus implying their involvement in inflammatory psoriatic manifestations. The altered expression of the five miRNAs in the peripheral circulation found in our study may reflect their dysregulation in keratinocytes and/or T cells, which constitute the key cell types involved in the pathophysiology of the disease, as depicted in Figure 5. Much of the literature on these miRNAs has demonstrated specific targets and associated pathways involved in the dysregulation of psoriatic keratinocytes, albeit with limited knowledge of their T cell-centric role in disease development.

miR-215 is associated with multiple cellular processes, viz., proliferation, apoptosis, migration, invasion, and epithelial–mesenchymal transition, in various cancers (79, 80). In psoriasis, miR-215 is reported to be downregulated in the skin lesion tissues in humans and mouse models, with the functional role validated in a keratinocyte-specific study, wherein miR-215 is shown to target DYRK1A and dysregulate keratinocyte proliferation via EGFR signaling (6, 52, 81). In the context of T cells, miR-215 has been reported to be differentially expressed in the Th2 subset in healthy human subjects, with no T cell-associated report in psoriasis (30, 82). IL-17 receptors, RUNX1, and IL-21 with miR-215-binding UTRs constitute Th17 subset-specific signature molecules, implying the potential role of miR-215 in psoriasis-associated Th17 dysregulation (62, 64, 65). Also, not a single report on expression patterns in peripheral circulation in psoriasis patients is available on miR-215, possibly due to its low abundance and the downregulated expression detected in our study. miR-148a has been demonstrated to contribute toward the maintenance of a chronic inflammatory immune-environment by regulating the persistence of activated Th1 cells in the murine helper T cell model (83). With upregulation in its expression in PBMCs from psoriasis patients, miRNA-148a has been shown to facilitate differentiation of inflammatory monocyte-derived dendritic cells via PU.1–miR-148a–MAFB axis, albeit with no report in the context of psoriatic lesions, Keratinocytes, T cells, or peripheral circulation (84). miR-223 is another miRNA associated with pathogenic T cells in autoimmune diseases like rheumatoid arthritis and multiple sclerosis (85–87). In the context of psoriasis, miR-223 is reported as one of the important miRNAs altered in skin lesions with changes in epidermal and dermal infiltrate along with peripheral Th17 subsets involved in the inflammatory disease outcome (7, 81, 88). Mechanistically, miR-223 has been shown to mediate keratinocyte hyperproliferation and apoptosis via the PTEN/Akt pathway in a HaCaT cell line model (48). Additionally, miR-223 is shown to be differentially upregulated in PBMCs, with variable reports on extracellular systemic circulation in psoriasis patients. Lovendorf et al. and Pivarcsi et al. reported no change in peripheral circulation in two independent reports, while Garcia et al. demonstrated an increase similar to our finding in psoriatic plasma samples (20, 26, 89). miR-142-3p is known to be a prominent hematopoietic miRNA with a role in T cell cycling and involvement in controlling T cell subset cAMP levels, with functional implications for Treg suppressor function (67, 90). The miRNA is shown to be differentially detected in the psoriatic skin miRNAome with differential expression in the psoriatic epidermis, dermal infiltrate, and peripheral T cells in the same patient cohort (7, 81, 88). Mechanistically, miR-142-3p has been shown to dysregulate keratinocyte proliferation and apoptosis via targeting Sema3A based on HaCAT cell line studies (53). Blood-based studies on miR-142 exhibit variable findings with no change to a downregulated expression pattern, unlike the significant increase found in plasma samples in our study (25, 26). miR-125b constitutes a signature miRNA responsible for the maintenance of T cell naivety and thus regulates effector T cell function based on its high expression in naive T cells in healthy individuals and naive T cells from psoriatic patients (30, 88). Lesional miRNA profiling is also reported with miR-125b as one of the most downregulated miRNAs in the epidermal layer and in dermal infiltrates (6, 88). The major cell type in psoriatic lesions with decreased expression is reported to be keratinocytes that exhibit miR-125b-mediated hyperproliferation and abnormal differentiation via multiple signaling pathways validated in *in vitro* studies (43, 54, 57, 91). Studies on serum expression patterns demonstrate different findings, with downregulation reported by Koga et al. and Pan M et al. and no change reported by Hernandez et al., unlike the significant upregulation detected in our study (19, 25, 54).

All five miRNAs, with their predicted or validated targets, constituted regulators of one or more immune inflammation-associated pathways, such that changes in their levels in keratinocytes and/or T cells can potentially lead to the development of autoimmune-psoriatic manifestations. The miR–mRNA network analysis with predicted/validated targets highlights the dysregulation of multiple autoimmune disease-related pathways that may contribute to keratinocyte hyperproliferation and abnormal differentiation, along with altered T cell activation, differentiation, and effector function. All the pathways, namely Wnt, MAPK, TGF-β, PI3K, mTOR, Notch, IL-21, IL-17, chemokine signaling, and TCR signaling, have been reported to directly impinge on activation and proliferative capabilities of keratinocytes and T cells with the elaboration of an inflammatory milieu as the result in disease development (41, 42, 45, 47, 51, 55, 56, 58, 61, 63). Based on our findings, much exploration of the downstream miRNA mechanisms involving multiple predicted targets still needs to be explored for their role in psoriasis.

Importantly, the five miRNAs as a promising disease diagnostic panel, along with their robust correlation with disease severity, corroborate the involvement of multiple miR-target pathways that may drive the disease. In the context of specific limitations in our study, each of the five miRNAs exhibited a significant correlation with only one or two of the other miRNAs in the panel. This could be because of the heterogeneous expression of different miRNAs in both healthy and psoriasis patients. An extended cohort of control and diseased subjects with the inclusion of more severe psoriasis patients may reveal a better relationship among the five miRNAs. Importantly, experimental validation of the downstream miRNA targets can further provide proof of concept for our *in silico*-derived miRNA–mRNA regulatory network. Nonetheless, in light of the existing literature, the plasma miRNAs, viz., miR-215, miR-142-3p miR-223, miR-125b-5p, and miR-148a, constitute a promising panel of miRNA-based biomarkers potentially involved in a pathogenic inflammatory response as a result of a dysregulated keratinocyte–T cell crosstalk.

## Data availability statement

The original contributions presented in the study are included in the article/Supplementary material, further inquiries can be directed to the corresponding author.

## Ethics statement

The studies involving human participants were reviewed and approved by the Ethics Committee of Guru Gobind Singh Medical College and Hospital, Faridkot, Punjab, India (GGS/IEC/19/57), AIIMS, Bathinda, India (IEC/AIIMS/BTI/150), and Central University of Punjab, Bathinda, Punjab, India (CUPB/IEC/2016/045). The patients/participants provided their written informed consent to participate in this study. Written informed consent was obtained from the individual(s) for the publication of any potentially identifiable images or data included in this article.

## Author contributions

PM and MJ conceived the original idea, planned the experiments, and wrote the manuscript. PM carried out the experiments. BB and SB provided the clinical samples and patient information. HSK provided support for the statistical analysis. NT and HRK helped with the miRNA-target network image. US and AJ critically revised the manuscript. MJ supervised and supported the research. All authors contributed to the article and approved the submitted version.
