# Supplementary material for: A panel of blood-based circulatory miRNAs with diagnostic potential in patients with psoriasis
Source: Front Med (Lausanne). 2023 Aug 28;10:1207993. doi: 10.3389/fmed.2023.1207993 (PMC10493330; doi:10.3389/fmed.2023.1207993)
Supplement: Supplementary file 1 [file Table_1.DOCX]

**Supplementary Table 1** Expression profiling of T cell associated miRNAs in plasma samples of Psoriasis vulgaris patients vs Healthy controls

| **Sr. No** | **miRNA** | **Sequence** | **Expression pattern** | **Functional Role** | **References** |
| --- | --- | --- | --- | --- | --- |
|  | miR-215-5p | AUGACCUAUGAAUUGACAGAC | Downregulation  (FCH= -2.24, *p-value*=0.043) | Associated with Th2 subset  Suppress IL-21 and targets IL17RA, IL17RE | (30, 69, 72) |
|  | miR-148a | UCAGUGCACUACAGAACUUUGU | Upregulation  (FCH= 1.82, *p-value*=0.032) | Regulates Th1 survival | (83) |
|  | miR-125b | UCCCUGAGACCCUAACUUGUGA | Upregulation  (FCH=1.84, *p-value*=0.034) | Maintain T cell naivity | (30) |
|  | miR-142-3p | UGUAGUGUUUCCUACUUUAUGGA | Upregulation  (FCH=2.56, *p-value*=0.045) | Regulate T Cell proliferation | (29, 78) |
|  | miR-223 | UGUCAGUUUGUCAAAUACCCCA | Upregulation  (FCH=2.42, *p-value*=0.028) | Modulate T cell differentiation | (85-87) |
|  | miR-146a | UGAGAACUGAAUUCCAUGGGUU | Upregulation(ns)  (FCH=1.82, *p-value*=0.36) | Regulates T cell activation | (28) |
|  | miR-21 | UAGCUUAUCAGACUGAUGUUGA | Upregulation (ns)  (FCH=1.84, *p-value*=0.48) | Regulates activation of CD4^+^and CD8^+^ T cells | (28) |
|  | miR-155 | UUAAUGCUAAUCGUGAUAGGGGUU | No change (ns)  (FCH=1.2, *p-value*=0.54) | Regulates T cell activation  Inhibit Th2 polarization, promotes Th17 differentiation | (28) |
|  | miR-590-5p | GAGCUUAUUCAUAAAAGUGCAG | Undetected in few Samples | Promotes pathogenic Th17 differentiation | (32) |
|  | miR-15b | UAGCAGCACAUCAUGGUUUACA | Undetected in few samples | T cell naivity | (29) |
|  | miR-568 | AUGUAUAAAUGUAUACACAC | Undetected in few samples | Inhibits T cell activation | (31) |
|  | miR-150 | UCUCCCAACCCUUGUACCAGUG | Undetected in few samples | Regulates T cell development Modulate T cell activation, proliferation and apoptosis Promotes regulatory T cell differentiation Regulates CD8+T cell differentiation | (28, 34, 35) |
|  | miR-23b | UGGGUUCCUGGCAUGCUGAUUU | Undetected in few samples | Inhibits Th2 polarization | (28) |
|  | miR-27b | AGAGCUUAGCUGAUUGGUGAAC | Undetected in few samples | Induces Th17 differentiation | (28) |
|  | miR-184 | UGGACGGAGAACUGAUAAGGGU | Undetected in few samples | Regulates T cell activation | (33) |
